# Harnessing Emotions to Enhance Feedback in the Emergency Department

**DOI:** 10.1002/aet2.70102

**Published:** 2025-11-12

**Authors:** Alessandra Karam, Morgan O'Neill, Katie Lorenz, Linda Regan, Jeremy Branzetti, Michael Gisondi, Laura R. Hopson

**Affiliations:** ^1^ Department of Emergency Medicine University of Michigan Ann Arbor Michigan USA; ^2^ Johns Hopkins University School of Medicine Baltimore Maryland USA; ^3^ Department of Emergency Medicine Yale University School of Medicine New Haven Connecticut USA; ^4^ Stanford School of Medicine Stanford California USA

## Abstract

Feedback in graduate medical education is often felt to be inadequate, particularly in the Emergency Department where limited time and continuity hinder meaningful reflections. We introduced an emotion‐linked debriefing model at three emergency medicine residencies, using structured prompts to explore both positive and negative emotions experienced during shifts. This approach encouraged richer feedback conversations, deeper trainee engagement, and generation of specific, actionable learning goals. Emotion‐linked debriefing offers a practical strategy to enhance real‐time feedback and warrants further evaluation of its educational impact.

## 1 Background

Trainees and instructors in graduate medical education (GME) frequently report inadequate feedback [1]. While multiple feedback models exist, their effectiveness, particularly for real‐time feedback, is often limited [2]. This challenge is exacerbated in the Emergency Department (ED) because of brief trainee‐instructor interaction, lack of schedule alignment, and absence of prior relationships [1, 2]. Models like R2C2 offer a valuable longitudinal approach to feedback, yet a key barrier remains: the structure to effectively engage in reflective feedback conversations centered on a detailed recollection of recent clinical experiences [1, 3].

Emotions play a crucial role in learning and memory [4]. They influence how trainees interpret and utilize feedback, as well as their motivation and performance. Information retrieval and learning are mediated by both positive and negative emotions [5]. Control Value Theory postulates that both negative and positive emotions can have activating effects on learning [4]. Prior research demonstrated that Master Adaptive Learners frequently use emotions—both positive and negative—triggered by the clinical environment to motivate their learning [6].

We propose that connecting feedback in the clinical learning environment to the emotions experienced by GME trainees may foster richer, more authentic feedback conversations by enhancing specificity and recall. A practical approach to this includes using questions specifically targeting emotional activation, such as “What caused you anxiety or feelings of unease during your shift today?” We follow this by collaboratively developing an actionable plan for learning to help mitigate or reinforce those emotions in future encounters.

## 2 Explanation

**FIGURE 1:**
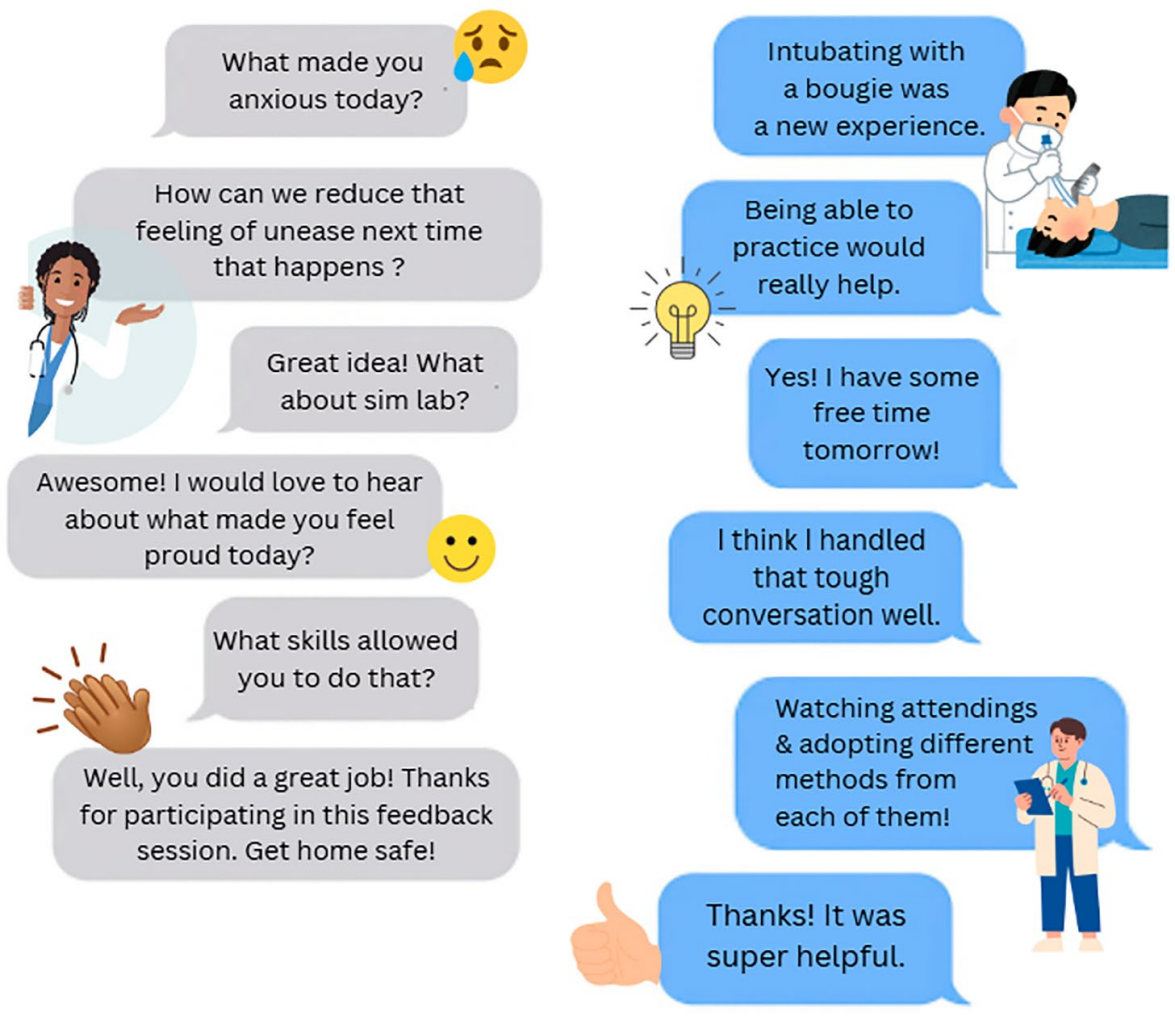
Example of emotion‐linked debriefing approach. The instructor (gray bubbles) explores an activating negative emotion (e.g., anxiety, stress, frustration) to prompt reflection by the trainee (blue bubbles) on clinical encounter(s) related to areas for development, then uses follow‐up questions to identify learning goals and create an actionable plan. The follow up question series uses an activating positive emotion (e.g., pride, excitement, accomplishment) which focuses on eliciting reflections of clinical encounters in which the trainee felt accomplished and reinforcing key underpinning skills.

Medical education faculty at three University‐based EM residency programs implemented these techniques during post‐shift feedback with trainees using a 4‐step question series (Figure 1). Faculty and trainees debriefed the clinical experience in a safe physical space using prompts focusing on both positive and negative activating emotions to elicit reflections on clinical performance from trainees. Follow‐up questions engaged the trainee to identify key skills and learning goals based on their reflections. For example, a trainee identifying that they felt anxious during airway management could be led into a discussion of what additional knowledge, skills, or experiences would help to ameliorate that feeling. Feedback through debriefing discussions from participants indicated richer conversations, increased engagement, and improved learner‐generated actionable learning items.

## 3 Description

Outcomes reported from the three EM residency sites implementing emotion‐linked debriefing with verbal feedback demonstrated advantages to utilizing this technique. Perceived advantages included more trainee engagement during feedback sessions as reflected by longer time spent engaging in this activity, and more specific actionable items generated by learners during feedback. Trainees largely responded positively to this technique and noted feeling motivated to take action using concrete insights into their performance. Importantly, a few learners found it challenging to engage in the call to emotional responses indicating the importance of tailoring this approach and the language used to the individual. As with all forms of feedback, the effectiveness of this technique is contingent on a psychologically safe learning environment and a trusting relationship [1]. Future work will determine if this model shows quantifiable value for trainee engagement and actionable learning items.

## Conflicts of Interest

The authors declare no conflicts of interest.

## Data Availability

Data sharing not applicable to this article as no datasets were generated or analysed during the current study.
